# Milk consumption in relation to incidence of nasopharyngeal carcinoma in 48 countries/regions

**DOI:** 10.1186/s12885-015-2021-3

**Published:** 2015-12-21

**Authors:** Zhi-Ming Mai, Ching-Man Lo, Jun Xu, King-Pan Chan, Chit-Ming Wong, Maria Li Lung, Tai-Hing Lam

**Affiliations:** School of Public Health, Li Ka Shing Faculty of Medicine, The University of Hong Kong, 5/F, William MW Mong Block, 21 Sassoon Road, Hong Kong S.A.R., China; Department of Clinical Oncology and Center for Cancer Research, The University of Hong Kong, Hong Kong S.A.R., China; Center for Nasopharyngeal Carcinoma Research (CNPCR), Research Grants Council Area of Excellence Scheme, The University of Hong Kong, Hong Kong S.A.R., China

**Keywords:** Milk consumption, Nasopharyngeal carcinoma, Ecological study, 48 countries/regions

## Abstract

**Background:**

Decreasing trends of nasopharyngeal carcinoma (NPC) incidence have been consistently reported in endemic populations but the etiology of NPC remains unclear. The objective of our study was to assess the international and local (Hong Kong) correlations of milk and dairy products per capita consumption with NPC incidence.

**Methods:**

We conducted an ecological study in 48 countries/regions. Age standardized incidence rates of NPC were obtained from the Cancer Incidence in Five Continents. Dairy product consumption and Human Development Index were obtained from the Food and Agriculture Organization of the United Nations and the United Nations Development Programme. Spearman correlation, multivariate analysis and time-lagged analysis were performed.

**Results:**

The negative correlations between milk consumption and decreased age standardized incidence rates of NPC were observed in the 48 countries/regions adjusting for Human Development Index in endemic countries/regions. In Hong Kong, multivariate analysis, after adjusting for other potential confounders, including salted fish, cigarette, vegetable consumption and socioeconomic status, showed consistently negative and significant correlations between milk consumption and NPC incidence (The strongest coefficient (β) was observed at 10-year lag in males [β = −0.439; *P* < 0.01] and in females [β = −0.258; *P* < 0.01]).

**Conclusions:**

Our study showed the correlations on milk consumption per capita and against lower risk of NPC in 48 countries/regions and in Hong Kong. These hypothesis-generating results could support further studies on individual exposures and the disease.

## Background

Nasopharyngeal carcinoma (NPC) is rare worldwide, but much more common in Southeast Asia [1]. One of the highest age-standardized incidence rates (ASIRs) of NPC was observed in Hong Kong (12.5 per 10^5^ person-years in males, 2012) [2], and similar ASIRs were observed in nearby cities including Zhongshan and Guangzhou, southern China [3]. By contrast, the ASIRs for most part of the world are less than 0.5 per 10^5^ person-years [3]. In addition to the marked geographic incidence pattern of NPC, decreasing trends of NPC incidence have been consistently reported in endemic populations [4]. In Hong Kong, for example, NPC was the 8^th^ most common cancer in 2012; however, the ASIR in males and females combined decreased by 68 % during 1983 to 2012 [2]. The endemic regions of NPC were marked by rapid economic growths and these growths might be associated with the observed declines [5], but the underlying causes are unclear.

Economic development may have a link with the changes of lifestyle, including decreased consumption of preserved foods and increased consumption of various food [5]. Cantonese-style salted fish was a staple preserved foods in southern China because they were cheap. It is considered as a major NPC risk factor and has been rated as a Group 1 carcinogen by the International Agency for Research on Cancer [6]. However, our recent ecological study did not find a correlation between the decline in salted fish consumption per capita and the corresponding changes in NPC incidence in 8 regions with varied NPC risk, including Hong Kong which has the highest risk [7]. The decreasing trend of NPC incidence may be due to other risk or protective factors.

The World Cancer Research Fund suggests that approximately 30–40 % of cancer cases are potentially preventable through changes in food consumption patterns [8]. Milk consumption has greatly increased in Asia, though is still far lower than Western countries [9]. Differences in milk consumption and in the incidence of NPC among populations worldwide are distinct. The limited evidence of milk consumption and NPC came from case-control studies, and majority of these in endemic regions were conducted two decades ago before the changes of dietary patterns with rapid economic development. The correlation between milk consumption and NPC remains unclear, and new evidence is needed. Case-control or cohort studies take some years to deliver results and should be conducted to test the hypothesis generated by ecological studies. Ecological study is the most cost-effective approach to assess the population disease impact from changes over several decades in the consumption of food items that are suspected to be harmful or protective, and can provide quick albeit preliminary evidence at low cost. A notable example is that the hypothesis of the correlation between higher milk consumption and increased risk of prostate cancer was generated from ecological studies [10], and has been later confirmed by further studies (case-control and cohort studies, and meta-analyses) which provide stronger evidence [11].

We therefore assessed the international and local (Hong Kong) correlations of milk and dairy products per capita consumption with NPC incidence.

## Methods

A total of 48 countries/regions with comprehensive and reliable cancer and food statistics data were included.

### NPC incidence statistics

ASIR for NPC (C11, ICD-10: The International Classification of Diseases 10th Revision) were obtained from Cancer Incidence in Five Continents, published by the International Agency for Research on Cancer, 1998–2002 (Vol. IX) [3]. All incidence data were adjusted to the Segi standard population [12]. The data collection procedures are shown in Appendix 1.

### Milk and dairy product consumption per capita in 48 countries/regions

Per capita milk consumption data from 1968 to 2002 were obtained from two international sources. Food supply data, including those for “milk (whole)”, “cheese”, “butter and ghee”, and “fresh cream”, were obtained from the Food Supply database maintained by the Food and Agriculture Organization of the United Nations (FAO) (http://faostat.fao.org/). Milk excluding butter per capita consumption was estimated from import, export, and change in stock volumes provided in the Food Balance Sheet of the FAO according to the formula in the International Farm Comparison Network dairy report 2004 [(import − export − change in stock) ÷ total population of the different countries/regions]. The 1950–2010 population data were obtained from the World Population Prospects: the 2012 Revision (http://esa.un.org/wpp/unpp/panel_population.html) [13].

### Potential confounders

Indicators of the social economic status of the 48 countries/regions were obtained from the Human Development Index (HDI) published by the United Nations, which includes national income, education and life expectancy.

### Salted fish, tobacco, vegetables consumption per capita, and Gross Domestic Product (GDP) per capita in multivariate analysis

Hong Kong has the highest ASIR among the 48 countries/regions, and has data on other risk factors. The data collection procedures have been described previously [7]. Briefly, per capita consumption of salted fish, tobacco and vegetables was estimated using the Service Centre on Trade Statistics, Census and Statistics Department, Hong Kong Special Administrative Region (HKSAR) Government, the Hong Kong Council on Smoking and Health via the General Household Survey and Thematic Household Survey 2008, and the Department Annual Report of the Agriculture, Fisheries and Conservation Department (procedures included in Appendix 2). The per capita GDP was obtained from the 2009 Gross Domestic Product (Yearly), Census and Statistics Department, HKSAR Government.

### Statistical analysis

Spearman correlation analysis was used to examine the correlation between the ASIR of NPC and milk and dairy product consumption per capita (1998–2002). Six additional sets of correlation were calculated for consumption with the following time lags: 5-year (1993–1997), 10-year (1988–1992), 15-year (1983–1987), 20-year (1978–1982), 25-year (1973–1977) and 30-year (1968–1972).

Regression analysis was used to examine the correlation of milk consumption with the log NPC incidence adjusting for HDI in order to comply with the assumptions underlying outliers. In our analysis,

$$ \mathrm{Log}\left(\mathrm{ASIR}\ \mathrm{of}\ \mathrm{N}\mathrm{P}\mathrm{C}\right)={\upbeta}_0+{\upbeta}_1{\left( milk\ \&\  dairy\  product\right)}_i+{\upbeta}_2(HDI) $$

where β_0_ is the intercept, β_1_ and β_2_ represent parameters in the multiplicative model for each milk and dairy products, and (*Milk* & *dairy product*)_*i*_denotes each type of milk and dairy product, *i* from 1 to 5.

Each histopathological diagnosis of NPC has its unique epidemiologic profile: keratinizing squamous carcinoma is the main histological subtype in low-risk areas, while non-keratinizing undifferentiated carcinoma is the predominant subtype in endemic areas [14]. Countries/regions were stratified by low- and high-risk countries/regions of NPC to indirectly examine the correlation between milk consumption and histology-specific NPC incidence according to the GLOBOCAN 2012 [15] which showed the countries/regions with the 20 highest ASIR of NPC in males in the world. Eight countries/regions in our analysis, including Hong Kong (ASIR of NPC: 17.82/10^5^ per person-years), Malaysia (11.91/10^5^), Mainland China (11.65/10^5^), Singapore (10.92/10^5^), Philippines (5.66/10^5^), Algeria (5.08/10^5^), Tunisia (4.28/10^5^) and Thailand (2.94/10^5^) were among the 20 countries/regions with the highest ASIR of NPC (Appendix 3). Our literature review showed that non-keratinizing undifferentiated carcinoma are the predominant subtype in these 8 high-risk countries/regions. The other 40 countries/regions with keratinizing squamous carcinoma as the predominant subtype were classified as low-risk (Appendix 4).

More detailed analysis was conducted for Hong Kong, which has the highest NPC risk and has reliable NPC and risk factors data. Multivariate linear regression analysis was performed between milk excluding butter (which was the only dairy item with available consumption data) consumption per capita and NPC incidence in Hong Kong, with adjustment for other recognized NPC risk factors (salted fish, cigarette smoking, vegetables consumption and socioeconomic status). Repeated analysis using various time lags (range: 0, 5, 10 years) of milk excluding butter consumption per capita prior to the ASIR of NPC was performed to test the robustness of estimates based on different assumptions in the latency period of cancer development subsequent to relevant exposures.

All analyses were performed using SPSS 20.0. *p* < 0.05 was considered statistically significant.

Ethics approval was not required as data in our study were secondary data from *Cancer Incidence in Five Continents series* published by the International Agency for Research on Cancer (http://ci5.iarc.fr/) and the Food Supply database maintained by the Food and Agriculture Organization of the United Nations (FAO) (http://faostat.fao.org/). These data were not directly collected from humans or animals.

## Results

### Correlation between NPC incidence and milk consumption in 48 countries/regions

Spearman rank correlation coefficients (ρ) for the a) cross-sectional and b) time-lagged analysis of the ASIR of NPC by different milk and dairy products are shown in Table 1.

**Table 1:** Spearman correlation coefficients in cross-sectional analysis and time-lagged analysis between the ASIR of NPC and milk and dairy consumption in 48 countries/regions, 1968–2002

		Cross sectional	Time-lagged (consumption period)					
		1998–2002	1993–1997 (5-year lag)	1988–1992 (10-)	1983–1987 (15-)	1978–1982 (20-)	1973–1977 (25-)	1968–1972 (30-)
Milk excluding butter (MEBC) (*N* = 48)	Males	−0.55***	−0.54***	−0.53***	−0.53***	−0.54***	−0.54***	−0.56***
	Females	−0.57***	−0.55***	−0.54***	−0.55***	−0.55***	−0.55***	−0.56***
Milk, whole (MWF) (*N* = 46)	Males	−0.35*	−0.44**	−0.45**	−0.46**	−0.45**	−0.47**	−0.52***
	Females	−0.29*	−0.40**	−0.43**	−0.46**	−0.45**	−0.47**	−0.51***
Cheese (CHF) (*N* = 43)	Males	−0.40**	−0.40**	−0.36*	−0.33*	−0.34*	−0.36*	−0.37*
	Females	−0.42**	−0.40**	−0.37*	−0.31*	−0.32*	−0.30*	−0.29
Butter & Ghee (BGF) (*N* = 43)	Males	−0.36*	−0.29	−0.32*	−0.30*	−0.33*	−0.35*	−0.40**
	Females	−0.44**	−0.36*	−0.38*	−0.38*	−0.39*	−0.40**	−0.43**
Fresh Cream (CRF) (*N* = 30)	Males	−0.53**	−0.65***	−0.71***	−0.68***	−0.68***	−0.65***	−0.65***
	Females	−0.63***	−0.71***	−0.75***	−0.72***	−0.67***	−0.62***	−0.58***

*ASIR* age standardized incidence rate, *NPC* nasopharyngeal carcinoma

*p < 0.05; **p < 0.01; ***p < 0.001 (2-tailed)

#### Cross-sectional analysis from 1998 to 2002 (Table 1)

The strongest negative correlation was observed between the ASIR of NPC and fresh cream (CRF) in females (ρ = −0.63; *p* < 0.001). The second strongest significant, but moderately negative, correlation was observed in milk, excluding butter (MEBC) in males (ρ = −0.55; *p* < 0.001). In addition, negative and significant correlations were also observed in the categories of milk (whole) (MWF), cheese (CHF), and butter and ghee (BGF), with Spearman coefficients of − 0.35 in males and −0.29 in females,−0.40 in males and −0.42 in females, and −0.36 in males and −0.44 in females, respectively.

#### Time-lagged analysis in seven lagged models (Table 1)

The strongest negative correlations between CRF and NPC were shown in a 10-year lagged model in males (ρ = −0.71; *p* < 0.001) and in females (ρ = −0.75; *p* < 0.001). The significant coefficients increased from the 5-year lag, were maximal at the 10-year lag in males and in females, and declined to the lowest values at the 30-year lag. MEBC, MWF, and BGF had similar patterns for their negative and significant correlations, where the Spearman coefficients increased monotonically. CHF showed a U-shape with significant coefficients in males, which became minimal at the 15-year lag in males and increased and peaked at the longest lag, but the pattern was unclear in females.

**Fig. 1:**
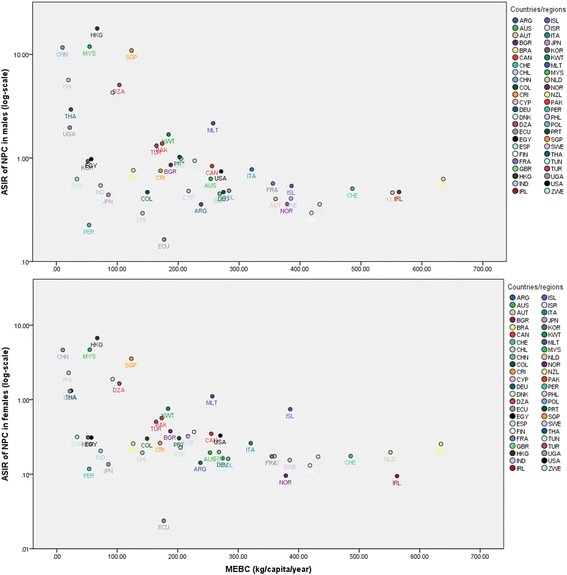
Correlation between ASIR of NPC per 10^5^ person-years and per capita consumption of milk excluding butter (MEBC) in 48 countries/regions in males (*top*) and in females (*bottom*), 1998–2002

Figure 1 shows the negative nonlinear correlation between the ASIR of NPC and per capita consumption of milk excluding butter (MEBC) in 48 countries/regions. The other dairy items showed similar patterns (figures not shown, but are included in Appendix 5). The ASIR of NPC ranged from 0.16/10^5^ person-years (Ecuador) to 17.82/10^5^ (HKSAR) in males (>100-fold difference), and from 0.02/10^5^ (Ecuador) to 6.72/10^5^ (HKSAR) in females (>300-fold difference). Per capita consumption of milk excluding butter varied markedly across the world (>60-fold difference), from about 10 kg/capita/year in China Mainland with lower milk consumption to about 640 kg/capita/year in New Zealand (Appendix 6).

### Regression analysis

Table 2 shows that the standardized coefficients (sβ) were consistently significant and negative between different milk and dairy product consumption per capita and the ASIR of NPC without adjustment. The strongest negative and significant correlations were observed in MEBC (sβ^c^ = −0.499 in males, sβ^c^ = −0.500 in females; *p* < 0.001).

**Table 2:** Regression coefficients between milk and dairy product consumption per capita and the ASIR of NPC in males and in females in 48 countries/regions, 1998–2002

		Without adjustment	Adjusting for HDI
	Sex	Standardized coefficient (sβ^a^)	Adjusted standardized coefficient (asβ^b^)
MEBC (*N* = 48)	Males	−0.499***	−0.552**
	Females	−0.500***	−0.534**
MWF (*N* = 46)	Males	−0.436**	−0.344*
	Females	−0.438**	−0.344*
CHF (*N* = 43)	Males	−0.406**	−0.249
	Females	−0.367*	−0.208
BGF (*N* = 43)	Males	−0.354*	−0.251
	Females	−0.340*	−0.246
CRF (*N* = 30)	Males	−0.497**	−0.325
	Females	−0.491**	−0.311

*ASIR* age standardized incidence rate, *NPC* nasopharyngeal carcinoma

*MEBC* milk excluding butter, *MWF* milk (whole), *CHF* cheese, B*GF* butter & ghee, *CRF* fresh cream

*p < 0.05; **p < 0.01; ***p < 0.001 (2-tailed)

^*a*^ Crude

^*b*^ Adjusted for Human Development Index

After adjusting for HDI, the negative and significant correlations (adjusted standardized coefficients [asβ]) in MEBC (asβ^a^ = −0.552 in males, asβ^a^ = −0.534 in females; *p* < 0.01) and MWF (asβ^a^ = −0.344 in males, *p* < 0.05 and asβ^a^ = −0.344 in females; *p* < 0.05) were found consistently, though the negative correlations between per capita consumption of other dairy products and the ASIR of NPC were attenuated and no longer statistically significant.

### Correlation between NPC incidence and milk consumption in high- and low-risk regions

Table 3 shows that the standardized coefficients in high-risk and in low-risk regions with adjustment for HDI. Negative correlations were observed in high-risk regions, but were not statistically significant. In low-risk regions, negative but insignificant coefficients were also observed in different dairy products with adjustment except for CRF with asβ^l^ = −0.444 in males; *p* = 0.04, and −0.375 in females; *p* = 0.08 which was marginally significant.

**Table 3:** Regression coefficients between milk and dairy consumption per capita and the ASIR of NPC in high- and low-risk countries/regions, 1998–2002

		High-risk			Low-risk		
	Sex	*N* ^h^	Adjusted standardized coefficient (asβ^h^)	*p* value	*N* ^l^	Adjusted standardized coefficient (asβ^l^)	*p* value
MEBC	Males	8	−0.122	0.75	40	−0.125	0.54
	Females		−0.254	0.50		−0.029	0.52
MWF	Males	6	−0.349	0.66	40	−0.060	0.72
	Females		−0.356	0.64		−0.091	0.59
CHF	Males	6	−0.003	0.99	37	−0.022	0.92
	Females		−0.055	0.95		−0.066	0.77
BGF	Males	6	−0.140	0.82	37	−0.040	0.82
	Females		−0.145	0.81		−0.048	0.79
CRF	Males	4	−0.631	0.44	26	−0.444*	0.04
	Females		−0.662	0.40		−0.375	0.08

*ASIR* age standardized incidence rate, *NPC* nasopharyngeal carcinoma

*MEBC* milk excluding butter, *MWF* milk (whole), *CHF* cheese, *BGF* butter & ghee, *CRF* fresh cream

*p < 0.05 (2-tailed). Adjusted for Human Development Index

^h^ High-risk regions

^l^ Low-risk regions

### Multivariate analysis in Hong Kong

Table 4 shows that negative and significant correlations between per capita consumption of milk excluding butter and ASIR of NPC were observed in Hong Kong at three lagged periods in model 1 (adjusted for per capita consumption of vegetables, salted fish and cigarette smoking) and model 2 (model 1 with additional adjustments, for GDP). The strongest negative correlation in model 1 was observed at 10-year lag in males (the adjusted unstandardized coefficient [auβ] = −0.454; *P* < 0.05) and in females (auβ = −0.232; *P* < 0.01), and in model 2 at 10-year lag in males (auβ = −0.439; *P* < 0.01) and in females (auβ = −0.258; *P* < 0.01).

**Table 4:** Regression coefficients from the multivariate linear regression analysis of ASIR of NPC on milk excluding butter (per kg), and various adjustments in Hong Kong, 1983–2009

Model	Lagged period (Years)	Consumption variable	ASIR in males		ASIR in females	
			Adjusted unstandardized coefficients (auβ)	*p* value	Adjusted unstandardized coefficients (auβ)	*p* value
1	0 (*N* = 27)	Milk excluding butter	−0.349***	<0.001	−0.155***	<0.001
	5 (*N* = 22)		−0.391***	<0.001	−0.181***	<0.001
	10 (*N* = 17)		−0.454*	<0.05	−0.232**	<0.01
2	0 (*N* = 27)	Milk excluding butter	−0.419***	<0.001	−0.184***	<0.001
	5 (*N* = 22)		−0.370***	<0.001	−0.151**	<0.01
	10 (*N* = 17)		−0.439**	<0.01	−0.258**	<0.01

Model 1: adjusted for per capita consumption of vegetable, salted fish and cigarette smoking

Model 2: adjusted for per capita consumption of vegetable, salted fish, cigarette smoking, and per capita Gross Domestic Product

*ASIR* age standardized incidence rate, *NPC* nasopharyngeal carcinoma

*p < 0.05; **p < 0.01; ***p < 0.001 (2-tailed)

## Discussion

The present study showed negative and significant correlations between ASIR of NPC and milk consumption in 48 countries/regions. Countries/regions have different histopathological subtypes of NPC of varied etiology. This may be an important reason underlying the varying patterns of risk factors found across different countries/regions [16]. Our detailed analysis in 8 high-risk countries/regions with predominantly (over 85 %) Type 3 histopathological diagnosis of NPC (non-keratinizing undifferentiated carcinoma) showed negative but non-significant correlations between increased milk consumption and decreased NPC incidence adjusting for HDI. In Hong Kong with the highest NPC risk, we found negative and significant correlations after adjusting for additional potential confounders, including salted fish, cigarette, vegetables consumption and GDP per capita.

### Strengths and limitations

Our study had several strengths, including the most comparable and reliable data, a global perspective (48 countries/regions) and a more detailed analysis on Hong Kong data with predominantly (over 95 %) Type 3 of histopathological diagnosis of NPC (non-keratinizing undifferentiated carcinoma) adjusting for potential confounders.

However, it also had several limitations. Although the FAO data are a cost-effective source for ecological comparisons and the best available data for a specific period in most countries/regions, these data did not represent real consumption in individuals (only the milk and dairy products available in the food supply). The major limitation is the ecological fallacy due to unmeasurable individual exposures and confounders. There may be residual confounding. The factors that affect consumption at population level are not the same at individual level. At population level, there are many determinant of various nature and studies based on individual exposure factors are needed. We took account important and known factors by adjusting for the specific time-dependent covariates (socioeconomic status) in the global analysis, with additional adjustment of major confounders, including salted fish, cigarette and vegetables consumption and socioeconomic status for Hong Kong analysis. Race and histology of NPC, are important factors for NPC studies, and should be taken into account in ecological analysis, but race- and histology-specific incidence data are not available in most cancer registries, suggesting that race- and histology-specific incidence data should be collected routinely to enable more in-depth analysis. In addition, temporal sequence was unclear in the cross-sectional analysis to establish causality. Our time-lagged analysis was performed in 5-year to 30-year lagged periods. The results showed that negative and significant coefficients generally increased with longer time lag but with inconsistent patterns for different dairy products, warranting further investigations using longitudinal and time-series analysis for specific dairy products.

## Conclusions

We found negative and significant correlations between milk and dairy products per capita consumption and NPC incidence in 48 countries/regions and in Hong Kong. These hypothesis-generating results could support further studies on individual exposures and the disease.

## Figures and Tables

**Fig. 2:**
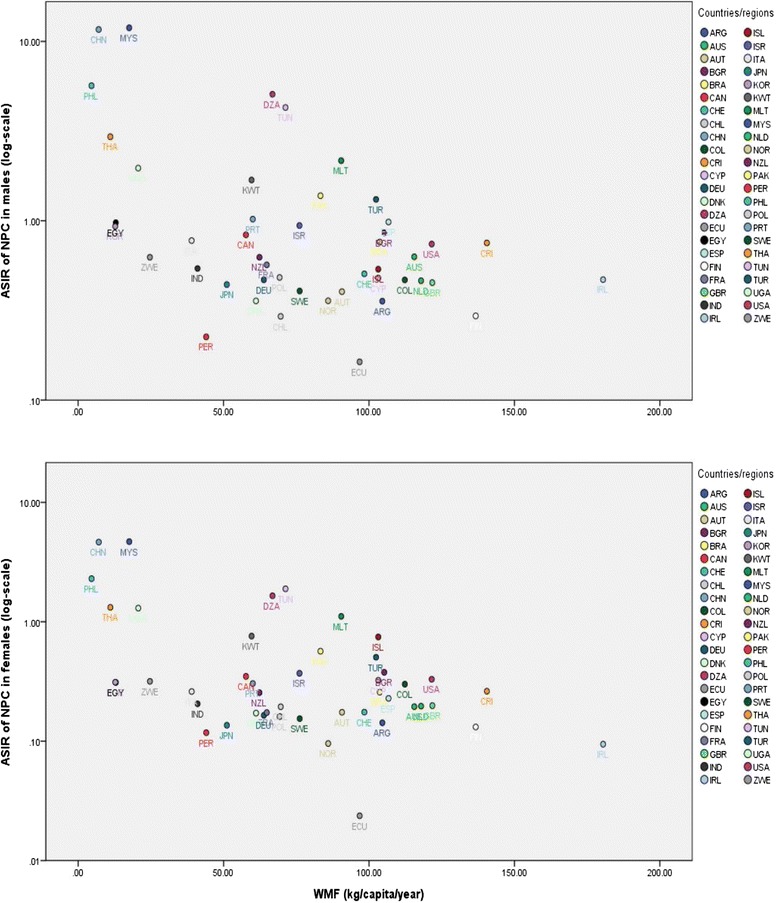
Relationships between ASIR of NPC per 10^5^ person-years and milk (whole) (WMF) in 48 countries/regions in males (*top*) and in females (*bottom*), 1998–2002. DZA: Algeria, EGY: Egypt, TUN: Tunisia, UGA: Uganda, ZWE: Zimbabwe, ARG: Argentina, BRA: Brazil, CHL: Chile, COL: Colombia, CRI: Costa Rica, ECU: Ecuador, PER: Peru, CAN: Canada, USA: USA, CHN: Mainland China, HKG: HKSAR, CYP: Cyprus, IND: India, ISR: Israel, JPN: Japan, KOR: Korea, KWT: Kuwait, MYS: Malaysia, PAK: Pakistan, PHL: Philippines, SGP: Singapore, THA: Thailand, TUR: Turkey, AUT: Austria, BGR: Bulgaria, DNK: Denmark, FIN: Finland, FRA: France, DEU: Germany, ISL: Iceland, IRL: Ireland, ITA: Italy, MLT: Malta, NOR: Norway, POL: Poland, PRT: Portugal, ESP: Spain, SWE: Sweden, CHE: Switzerland, NLD: Netherlands, GBR: United Kingdom, AUS: Australia, NZL: New Zealand

**Fig. 3:**
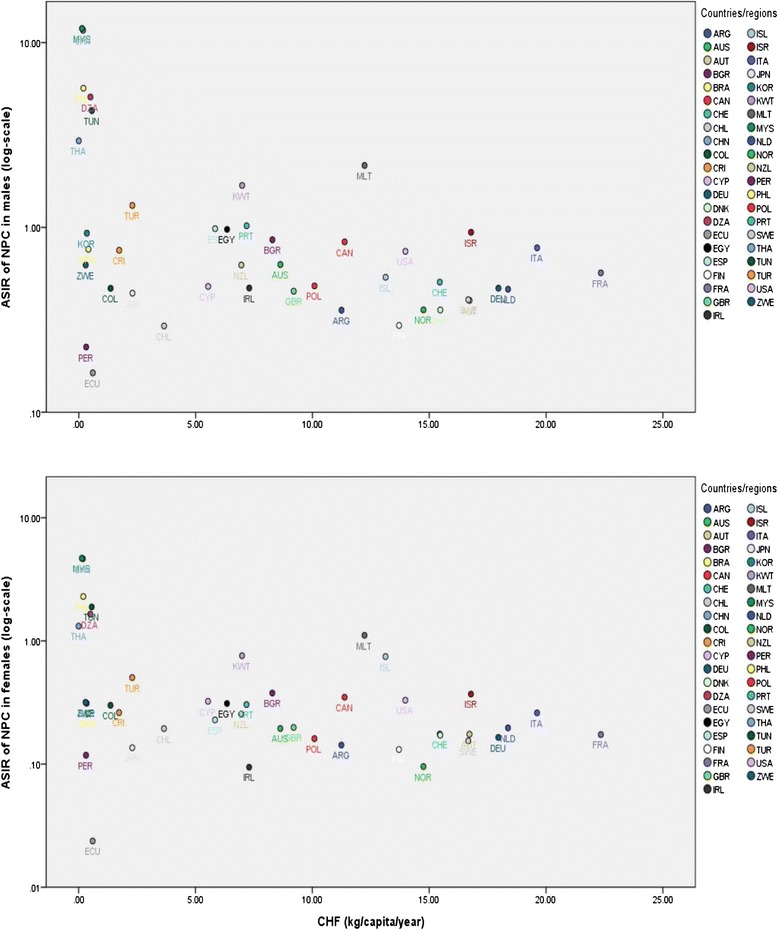
Relationships between ASIR of NPC per 10^5^ person-years and cheese (CHF) in 48 countries/ regions in males (*top*) and in females (*bottom*), 1998–2002. DZA: Algeria, EGY: Egypt, TUN: Tunisia, UGA: Uganda, ZWE: Zimbabwe, ARG: Argentina, BRA: Brazil, CHL: Chile, COL: Colombia, CRI: Costa Rica, ECU: Ecuador, PER: Peru, CAN: Canada, USA: USA, CHN: Mainland China, HKG: HKSAR, CYP: Cyprus, IND: India, ISR: Israel, JPN: Japan, KOR: Korea, KWT: Kuwait, MYS: Malaysia, PAK: Pakistan, PHL: Philippines, SGP: Singapore, THA: Thailand, TUR: Turkey, AUT: Austria, BGR: Bulgaria, DNK: Denmark, FIN: Finland, FRA: France, DEU: Germany, ISL: Iceland, IRL: Ireland, ITA: Italy, MLT: Malta, NOR: Norway, POL: Poland, PRT: Portugal, ESP: Spain, SWE: Sweden, CHE: Switzerland, NLD: Netherlands, GBR: United Kingdom, AUS: Australia, NZL: New Zealand

**Fig. 4:**
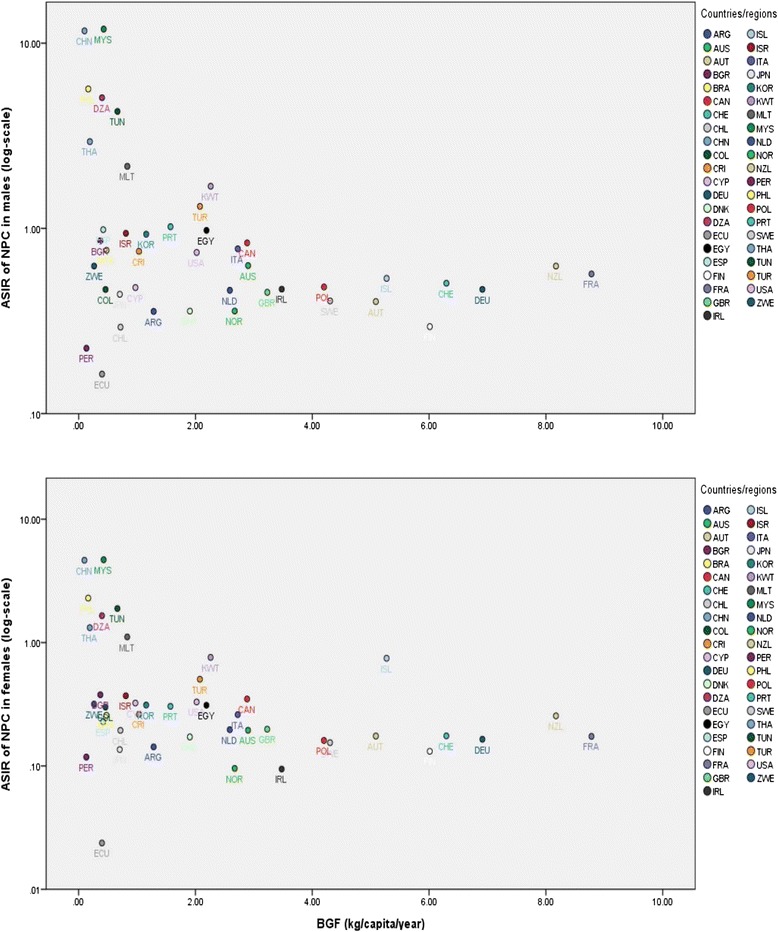
Relationships between ASIR of NPC per 10^5^ person-years and butter and ghee in 48 countries/regions in males (*top*) and in females (*bottom*), 1998–2002. DZA: Algeria, EGY: Egypt, TUN: Tunisia, UGA: Uganda, ZWE: Zimbabwe, ARG: Argentina, BRA: Brazil, CHL: Chile, COL: Colombia, CRI: Costa Rica, ECU: Ecuador, PER: Peru, CAN: Canada, USA: USA, CHN: Mainland China, HKG: HKSAR, CYP: Cyprus, IND: India, ISR: Israel, JPN: Japan, KOR: Korea, KWT: Kuwait, MYS: Malaysia, PAK: Pakistan, PHL: Philippines, SGP: Singapore, THA: Thailand, TUR: Turkey, AUT: Austria, BGR: Bulgaria, DNK: Denmark, FIN: Finland, FRA: France, DEU: Germany, ISL: Iceland, IRL: Ireland, ITA: Italy, MLT: Malta, NOR: Norway, POL: Poland, PRT: Portugal, ESP: Spain, SWE: Sweden, CHE: Switzerland, NLD: Netherlands, GBR: United Kingdom, AUS: Australia, NZL: New Zealand

**Fig. 5:**
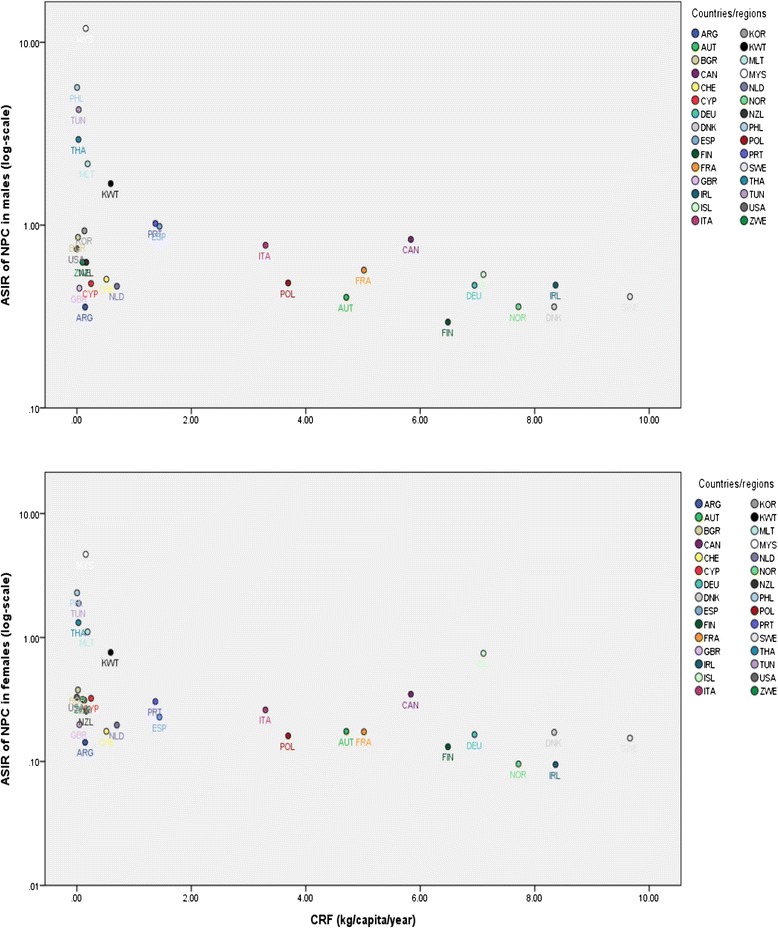
Relationships between ASIR of NPC per 10^5^ person-years and cream (CRF) in 48 countries/regions in males (*top*) and in females (*bottom*), 1998–2002. DZA: Algeria, EGY: Egypt, TUN: Tunisia, UGA: Uganda, ZWE: Zimbabwe, ARG: Argentina, BRA: Brazil, CHL: Chile, COL: Colombia, CRI: Costa Rica, ECU: Ecuador, PER: Peru, CAN: Canada, USA: USA, CHN: Mainland China, HKG: HKSAR, CYP: Cyprus, IND: India, ISR: Israel, JPN: Japan, KOR: Korea, KWT: Kuwait, MYS: Malaysia, PAK: Pakistan, PHL: Philippines, SGP: Singapore, THA: Thailand, TUR: Turkey, AUT: Austria, BGR: Bulgaria, DNK: Denmark, FIN: Finland, FRA: France, DEU: Germany, ISL: Iceland, IRL: Ireland, ITA: Italy, MLT: Malta, NOR: Norway, POL: Poland, PRT: Portugal, ESP: Spain, SWE: Sweden, CHE: Switzerland, NLD: Netherlands, GBR: United Kingdom, AUS: Australia, NZL: New Zealand

## Appendix 1

There were four categories of countries/regions according to the different methods of data collection using the CI5, Vol. IX:

- For the 16 countries (Australia, Brazil, France, Germany, India, Italy, Japan, China, Malaysia, Poland, Portugal, Spain, Switzerland, Thailand, Turkey, and United Kingdom), each with one or more cancer registries operating, the mean value of the ASIR from different cancer registries was calculated and used as the representative rate for the country. In Italy, for example, 22 ASIRs of NPC in 22 cancer registries were averaged, resulting in one mean value to represent the ASIR of NPC in Italy.
- For 12 countries (Algeria, Egypt, Tunisia, Uganda, Zimbabwe, Argentina, Chile, Colombia, Ecuador, Peru, Pakistan, and the Philippines), only one cancer registry was operating in each. The ASIR of the single cancer registry was used to estimate the NPC incidence for the country.
- The US has many cancer registries by different states. Among them, the registry for all racial groups in the Surveillance, Epidemiology, and End Results (SEER) Program of the National Cancer Institute (9 registries), which collected data in longer periods, was used as representative for the US.
- For the remaining 19 countries/regions (Costa Rica, Canada, Cyprus, Israel, Korea, Kuwait, Austria, Bulgaria, Denmark, Finland, Hong Kong Special Administrative Region (HKSAR), Iceland, Ireland, Malta, The Netherlands, Norway, Singapore, Sweden and New Zealand), the cancer registries collected data for the whole country/region. The incidence rates for these countries/regions were used as provided.

In some countries (Zimbabwe, Israel, and Kuwait), analysis by ethnic groups was supported by some cancer registries. In our analysis, Zimbabwe included the African race as the representative population, Israel included all ethnic groups (both Jews and non-Jews), and Kuwait included Kuwaitis.

In a separate analysis, annual NPC ASIRs in Hong Kong from 1983 to 2009 were obtained from the Hong Kong Cancer Registry with the 1966 world standard population as reference.

## Appendix 2

**Table 5:** Sources of data and years of data availability from 4 regions

Countries/regions	Age-standardized incidence rate	Salted fish consumption	Vegetable consumption	Cigarette consumption	Gross domestic product
Hong Kong	HKCR^†^, 1983−2009	SCTS^‡^, 1983−2009	AFCD^§^, 1983−2009	COSH^¶^, 1983−2009	HKCR^†^, 1983−2009

^†^ Hong Kong Cancer Registry

^‡^ Service Centre on Trade Statistics, Census and Statistics Department, Hong Kong Special Administrative Region Government

^§^ Agriculture, Fisheries and Conservation Department, Hong Kong

^¶^ Hong Kong Council in Smoking and Health

## Appendix 3

**Table 6:** Baseline characteristics of 8 high-risk countries/regions, 1998–2002

High-risk regions	ASIRM	ASIRF	MEBC	MWF	CHF	BGF	CRF
Algeria	5.08	1.65	111.44	72.42	0.60	0.34	-
Tunisia	4.28	1.89	101.12	79.14	0.56	0.50	0.14
China	11.65	4.65	11.64	8.40	0.18	0.10	-
HKSAR	17.82	6.72	67.53	-	-	-	-
Singapore	10.92	3.56	124.64	-	-	-	-
Malaysia	11.91	4.69	50.59	18.14	0.16	0.38	0.12
Philippines	5.66	2.29	19.18	4.28	0.20	0.12	0.02
Thailand	2.94	1.32	21.94	9.98	0.00	0.20	0.00
Average	8.78	3.35	63.51	32.06	0.28	0.27	0.07

*ASIRM* ASIR of NPC in males, *ASIRF* ASIR of NPC in females, *MEBC* milk excluding butter using International Farm Comparison Network report 2004, *MWF* whole milk collected from Food Balance Sheets, *CHF* cheese collected from Food Balance Sheets, *BGF* butter & ghee collected from Food Balance Sheets, *CRF* fresh cream collected from Food Balance Sheets

## Appendix 4

**Table 7:** Baseline characteristics of 40 low-risk countries/regions, 1998–2002

Low-risk regions	ASIRM	ASIRF	MEBC	MWF	CHF	BGF	CRF
Egypt	0.98	0.31	60.99	14.14	6.82	2.18	-
Uganda	1.97	1.30	22.68	21.44	-	-	-
Zimbabwe	0.63	0.32	36.22	27.18	0.30	0.24	0.10
Argentina	0.36	0.14	229.70	99.56	11.64	1.32	0.12
Brazil	0.76	0.26	126.82	105.34	0.34	0.48	-
Chile	0.29	0.19	138.64	65.50	3.66	0.80	-
Colombia	0.47	0.30	153.52	115.82	1.32	0.50	-
Costa Rica	0.75	0.26	180.46	148.02	1.90	1.18	-
Ecuador	0.16	0.02	208.97	92.18	0.60	0.40	-
Peru	0.23	0.12	53.05	42.78	0.34	0.20	-
Canada	0.84	0.35	254.72	55.48	11.70	2.86	6.36
USA	0.74	0.33	270.99	122.52	14.46	2.10	0.00
Cyprus	0.48	0.32	216.48	104.22	4.94	1.00	0.24
India	0.54	0.21	75.71	39.54	-	2.00	-
Israel	0.94	0.37	225.39	75.92	16.62	0.76	-
Japan	0.44	0.14	84.00	50.34	2.48	0.68	-
Korea	0.93	0.31	55.74	15.38	0.50	1.18	0.10
Kuwait	1.69	0.76	171.09	77.90	6.96	2.38	1.42
Pakistan	1.38	0.57	177.94	85.76	-	3.42	-
Turkey	1.32	0.50	151.31	94.50	2.04	1.86	-
Austria	0.40	0.17	350.64	83.12	19.14	5.06	3.92
Bulgaria	0.86	0.38	193.92	103.82	7.60	0.30	0.04
Denmark	0.36	0.17	442.34	52.92	16.02	1.68	9.14
Finland	0.29	0.13	417.16	132.52	14.80	5.26	6.44
France	0.57	0.17	356.50	63.24	23.36	8.68	5.40
Germany	0.47	0.16	278.57	62.38	18.80	6.70	7.32
Iceland	0.54	0.75	382.65	94.94	14.86	4.36	7.14
Ireland	0.47	0.09	605.87	190.88	8.90	3.10	8.10
Italy	0.78	0.26	322.29	41.58	21.42	3.02	3.42
Malta	2.16	1.11	266.57	88.98	12.66	0.46	0.12
Norway	0.36	0.10	360.72	77.42	14.84	2.90	7.52
Poland	0.48	0.16	284.82	64.36	11.72	4.40	4.08
Portugal	1.02	0.30	219.12	61.72	8.36	1.98	1.14
Spain	0.98	0.23	206.56	105.40	6.38	0.72	1.70
Sweden	0.41	0.15	386.71	79.48	16.98	3.70	9.82
Switzerland	0.51	0.18	479.55	95.46	16.86	6.10	0.58
Netherlands	0.46	0.20	529.64	119.28	20.14	2.76	0.78
UK	0.45	0.20	266.88	121.84	9.78	3.26	0.02
Australia	0.63	0.19	256.36	100.78	9.26	3.04	-
New Zealand	0.63	0.25	669.59	55.78	4.84	8.18	0.20
Average	0.72	0.31	254.27	81.24	9.82	2.59	3.28

*ASIRM* ASIR of NPC in males, *ASIRF* ASIR of NPC in females, *MEBC* milk excluding butter using International Farm Comparison Network report 2004, *MWF* whole milk collected from Food Balance Sheets, *CHF* cheese collected from Food Balance Sheets, *BGF* butter & ghee collected from Food Balance Sheets, *CRF* fresh cream collected from Food Balance Sheets

## Appendix 6

**Table 8:** Baseline characteristics of ASIR of NPC, HDI and milk & dairy products consumption per capita in 48 countries/regions, 1998–2002

Variable	Mean	SD	Variance	*N*
ASIR in males (per 10^5^)	2.06	3.64	13.27	48
ASIR in females (per 10^5^)	0.82	1.38	1.90	48
Human Development index (HDI)	0.83	0.12	0.02	48
Milk excluding butter (MEBC) (kg/capita/year)	222.48	157.98	24957.57	48
Milk, whole (MWF) (kg/capita/year)	74.82	40.03	1602.63	46
Cheese (CHF) (kg/capita/year)	8.49	7.21	51.86	43
Butter & Ghee (BGF) (kg/capita/year)	2.29	2.17	4.72	45
Fresh Cream (CRF) (kg/capita/year)	2.85	3.35	11.21	30

*ASIR* Age-standardized incidence rate

## Abbreviations

asβ: adjusted standardized regression coefficients
auβ: adjusted unstandardized regression coefficients
ASIR: age-standardized incidence rates
BGF: butter & ghee
CRF: fresh cream
CHF: cheese
FAO: Food and Agriculture Organization of the United Nations
HDI: Human Development Index
ICD-10: The International Classification of Diseases 10^th^ Revision
GDP: Gross Domestic Product
HKSAR: Hong Kong Special Administrative Region
MEBC: milk excluding butter
MWF: milk, whole
NPC: nasopharyngeal carcinoma
ρ: Spearman rank correlation coefficients
sβ: standardized regression coefficients
